# Regulatory T cells protected against abdominal aortic aneurysm by suppression of the COX‐2 expression

**DOI:** 10.1111/jcmm.14554

**Published:** 2019-07-21

**Authors:** Bin Liu, Jing Kong, Guipeng An, Kai Zhang, Weidong Qin, Xiao Meng

**Affiliations:** ^1^ Key Laboratory of Cardiovascular Remodeling and Function Research, Chinese Ministry of Education, Chinese Ministry of Health and Chinese Academy of Medical Sciences The State and Shandong Province Joint Key Laboratory of Translational Cardiovascular Medicine, Qilu Hospital of Shandong University Jinan China; ^2^ Department of Cardiology Qilu Hospital of Shandong University Jinan China; ^3^ Department of Critical Care Medicine Qilu Hospital of Shandong University Jinan China

**Keywords:** abdominal aortic aneurysm, cyclooxygenase‐2, immune treatment, regulatory T cells

## Abstract

CD4^+^CD25^+^ regulatory T cells (Tregs) have been shown to protect against the development of abdominal aortic aneurysm (AAA). Cyclooxygenase‐2 (COX‐2), a pro‐inflammatory protein, can convert arachidonic acid into prostaglandins (PGs). The present study was aimed to investigate the effect of Tregs on COX‐2 expression in angiotension II (Ang II)‐induced AAA in ApoE^−/−^ mice. Tregs were injected via tail vein in every 2 weeks. Ang II was continuously infused by a micropump for 28 days to induce AAA. In vivo, compared with the control group, adoptive transfer of Tregs significantly reduced the incidence of AAA, maximal diameter, and the mRNA and protein expression of COX‐2 in mice. Immunofluorescence showed that Tregs treatment reduced COX‐2 expression both in smooth muscle cells (SMCs) and macrophages in AAA. In vitro, the Western blot analysis showed that Tregs reduced Ang II‐induced COX‐2 expression in macrophages and SMCs. Meanwhile, ELISA showed that Tregs reduced Ang II‐induced prostaglandin E_2_ (PGE_2_) secretion. Moreover, Tregs increased SMC viability and induced transition of macrophages phenotype from M1 to M2. In conclusion, Tregs treatment dramatically decreased the expression of COX‐2 in vivo and in vitro, suggesting that Tregs could protect against AAA through inhibition of COX‐2. The study may shed light on the immune treatment of AAA.

## 1 INTRODUCTION

Abdominal aortic aneurysm (AAA), as a chronic vascular degenerative disease, is characterized with a progressive dilation and remodelling of the vessel wall, leading to a lethal risk of aortic rupture, especially in the elderly.^1^, ^2^ However, the pathogenesis of AAA is still not fully explained. Although there are some common features between AAA and atherosclerosis, the medical treatment for coronary artery disease is not available to reduce AAA formation or expansion in human, and currently the principle therapy still depends on surgical interventions.^3^ Recent evidence indicated that chronic inflammation played a critical role in the pathogenesis of AAA, and inhibition of inflammatory response may be a therapeutic approach for the prevention of AAA.^4^

Cyclooxygenase‐2 (COX‐2), a key regulator enzyme of prostaglandins (PGs) synthesis, catalyses the conversion process of arachidonic acids to PGs. COX‐2 modulates pro‐inflammatory chemokines expression, influences cell proliferation and functions.^5^, ^6^, ^7^ COX‐2 was normally undetectable in most human tissues, but it could be rapidly induced in response to inflammatory stimuli, such as lipopolysaccharide (LPS) or angiotensin II (Ang II).^8^, ^9^ The expression of COX‐2 is well‐characterized in chronic inflammatory disorders, such as atherosclerosis.^10^ Genetic knockout of COX‐2 resulted in significant attenuation of acute inflammation corresponding with decreased PGE_2_ levels.^11^

It has been demonstrated that immune system took part in the pathogenesis of many cardiovascular diseases.^12^ CD4^+^CD25^+^ regulatory T cells (Tregs), as an important component of the immune system, maintained immunological homeostasis and tolerance, and prevented excessive immune responses.^13^ An impaired function or a deficiency of Tregs can result in immune dysregulation and autoimmune disease.^13^ It has been documented that Tregs have a protective role in many cardiovascular disease, including atherosclerosis, hypertension, myocarditis and dilated cardiomyopathy.^14^ Recently, Tregs have been showed to prevent the development of Ang II‐induced AAA in mice.^15^, ^16^

Currently, little is known about the correlation between Tregs and COX‐2 in AAA. Therefore, by use of an AAA model, we attempt to investigate the potential mechanism and look for an immune treatment of AAA.

## 2 MATERIALS AND METHODS

### 2.1 Animal models and intervention

Animal experiments were approved by ethics committee of Shandong University and complied with the guidelines of the Animal Management Rules of the Chinese Ministry of Health. Ten C57BL/6J wild‐type mice (male, 8 weeks old) were obtained from the Beijing University Animal Research Center and used as donors of Tregs. A CD4^+^CD25^+^ Regulatory T Cell Isolation Kit (Miltenyi Biotec) was used to obtain Tregs from the splenocytes of C57BL/6J mice according to the manufacturer's instructions. Purified Tregs were suspended in phosphate‐buffered saline (PBS, 200 μL) for further injection.

Thirty male ApoE^−/−^ mice (3 months old) on a C57BL/6J background were housed in pathogen‐free condition and kept on a 12‐hour light/12 hour dark cycle. During the entire experimental period, all ApoE^−/−^ mice were fed with a high‐fat diet (0.25% cholesterol and 15% cocoa butter) and had full access to food and water. These mice were randomly divided into three groups (n = 10 per group): the control group (received no treatment), the PBS group (received an intravenous injection of PBS) and the Tregs group (received an intravenous injection of 10^6^ Tregs). One day after injection, Ang II (1000 ng/kg/min) was continuously infused for 28 days via a mini‐osmotic pump as described previously.^15^, ^17^ PBS and Tregs were injected repeatedly after 2 weeks. After 28 days of Ang II infusion, all mice underwent euthanasia and their aortic tissues were collected.

### 2.2 Histological analysis

Euthanized mice were perfused with saline to eliminate blood in the lumen, and the aortic arteries were removed and fixed in 4% paraformaldehyde. The formation of AAA was evaluated by measuring the maximum external diameter of abdominal aorta, which defined as at least 50% dilation.^2^ Then, the abdominal arteries were embedded in OCT compound and serial sections (5 μm) were cut for haematoxylin and eosin (H&E) staining or immunohistochemical staining to assess the expression of COX‐2 (1:500, Abcam). Positive staining areas of COX‐2 were digitally captured and calculated by a computer‐assisted automated image analysis system (Image‐Pro Plus 6.0, Media Cybernetics).

### 2.3 Immunofluorescence

After blocked with BSA for 30 minutes, sections (5 μm) of abdominal arteries were incubated with rabbit anti‐COX‐2 (1:100, Abcam), mouse anti‐CD68 (1:100, Abcam) or mouse anti‐α‐SM actin (1:100, Sigma‐Aldrich) overnight at 4°C. After incubated with secondary antibodies, a drop of Prolong Gold anti‐fade reagent with DAPI (Vector Laboratories) was used to seal the coverslip. Images were acquired by laser scanning confocal microscopy (LSM 710, Zeiss).

### 2.4 Cell co‐culture and treatment

In the first part of in vitro study, the RAW264.7 mouse macrophages cultured in DMEM medium (GIBCO) containing 10% foetal bovine serum, 100 U/mL penicillin and 100 g/mL streptomycin at 37°C in 5% CO_2_. Macrophages were randomly divided into three groups: the control group, the CD25^−^ group (CD4^+^CD25^−^ T cells, 5 × 10^5^) and the Tregs group (Tregs, 5 × 10^5^) for 48 hours with anti‐CD3 antibody (50 ng/mL). Then, cells were stimulated with Ang II (1 μmol/L) for 24 hours. Floating T cells were discarded, and macrophages were harvested for further analysis.

In the second part of in vitro study, mouse aortic smooth muscle cells (SMCs, ATCC) were cultured without T cells (control group) or with CD4^+^CD25^−^ T cells (CD25^−^ group, 5 × 10^5^) or mouse Tregs (5 × 10^5^) for 48 hours with anti‐CD3 antibody (50 ng/mL), followed by an additional stimulation with Ang II (1 μmol/L) for 24 hours. Floating T cells were discarded, and SMCs were harvested for further analysis.

### 2.5 ELISA

The supernatants of cell mediums were collected, and the concentration of PGE_2_ was assayed by use of an ELISA kit (BlueGene Biotech.) following manufacturer's instructions.

### 2.6 MTT

The viability of SMCs was determined by MTT assay (Beyotime). Briefly, SMCs were seeded into 96‐well plates at a density of 5000 cells/well. Following exposure to stimulation, SMCs were incubated with 10 µL MTT (5 mg/mL)/well reagent for 4 hours at 37°C. The supernatant was carefully removed, and 75 µL/well dimethyl sulphoxide (DMSO) was added to dissolve the formazan crystals. Samples were then analysed at 570 nm using a Varioskan Flash multifunction plate reader (Thermo Scientific).

### 2.7 Real‐time PCR

Total RNA was isolated from the abdominal aortic segments and the harvested cells by use of Trizol reagent (Invitrogen) according to the manufacturer's instruction. The cDNA was synthesized by reverse transcription of 1μg RNA by use of iScript cDNA synthesis kit (Bio‐Rad). RT‐PCR was performed to determine the mRNA expression of CD68, inducible nitric oxide synthase (iNOS), arginase‐1 (Arg‐1), chemokine ligand‐9 (CCL‐9), COX‐2 and β‐actin. The primers used in the study were shown in Table 1. The β‐actin was used as an internal control.

**Table 1:** The sequences of primers for real‐time PCR

Genes	Forward	Reverse
COX‐2	5′‐AAAGTTCAGCCATTGTACAGCAGG‐G‐3′	5′‐GAATCTCCTAGAACTGACTGG‐3′
CD68	5′‐CTGGACTCTACGACTTCACAATG‐3′	5′‐AGTTGGCGATCACTGACAGTT‐3′
iNOS	5′‐CCTTGCACTGCCAAGAATTTG‐3′	5′‐CATTGCGTCACTGGATAGTAGTT‐3′
Arg‐1	5′‐CTGAGAGATTCAAGGCAAGAGG‐3′	5′‐GAACGCGCTATCTTACCCCAG‐3′
CCL9	5′‐CCCTCTCCTTCCTCATTCTTACA‐3′	5′‐AGTCTTGAAAGCCCATGTGAAA‐3′
β‐actin	5′‐CACTGTGCCCATCTACG A‐3′	5′‐GTAGTCTGTCAGGTCCCG‐3′

Abbreviations: Arg‐1, arginase‐1; CCL‐9, chemokine ligand‐9; COX‐2, cyclooxygenase‐2; iNOS, inducible nitric oxide synthase.

### 2.8 Western blot analysis

Total proteins were extracted from the abdominal aortic segments or the cells. Equal amounts of protein samples were run on 10% SDS‐polyacrylamide gels and electrotransferred onto polyvinylidene difluoride (PVDF) membranes. After incubation in 5% skim milk for 2 hours at room temperature, the membranes were then incubated with a specific antibody against COX‐2 (1:500, Abcam) and β‐actin (1:1000, cell signalling technology) overnight at 4°C. Subsequently, the membranes were further incubated with horseradish peroxidase‐conjugated secondary antibodies after washed with Tris‐buffered saline with Tween 20 (TBST). At last, the blots were visualized by use of the enhanced chemiluminescence detection system (Pierce) according to the supplier's recommendations. Sample loading was normalized to β‐actin expression.

### 2.9 Statistical analyses

SPSS 15.0 (SPSS Inc) software was used for statistical analysis. Values were presented as mean ± SD, and One‐way ANOVA test with LSD post hoc analysis was used for multiple comparisons. *P* < .05 was considered statistically significant.

## 3 RESULTS

### 3.1 Tregs transfer attenuated AAA incidence in Ang II‐infused mice

**Figure 1:**
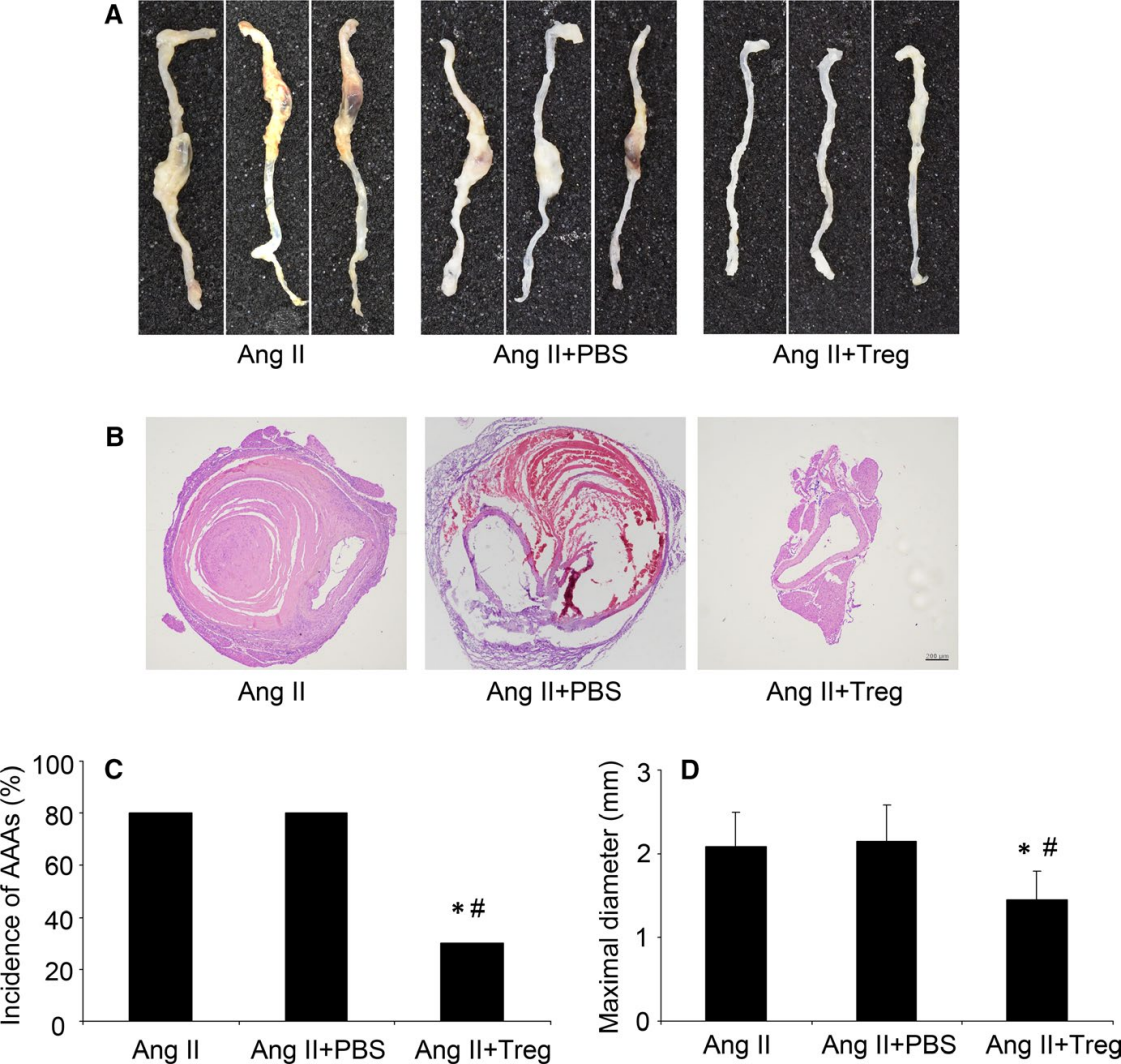
Tregs treatment reduced the formation of AAA in Ang II‐induced ApoE^−/−^ mice. A, Representative photographs of abdominal aortic specimens in three groups of mice; B, H&E staining of three groups of mice；Bar = 200 µm; C, Incidence of AAA in three groups of mice; D, Maximal abdominal aortic diameters in three groups of mice. Ang II, angiotension II. ^*^ *P* < 0.05 vs AngII group, ^#^ *P* < 0.05 vs AngII + PBS group

Chronic infusion of Ang II into ApoE^−/−^ mice has been widely used to induce AAA,^17^, ^18^ which was also used in the experiment. Consistent with prior studies, we found that Ang II infusion could successfully induce AAA in ApoE^−/−^ mice. As shown in Figure 1, the incidence of AAA was 80% (8/10) and 80% (8/10) in the control group and PBS group, respectively, while injection of Tregs (10^6^ cells) reduced the incidence of AAA to only 30% (3/10) (*P* < 0.05, Figure 1A and C). Moreover, the H&E staining showed that Ang II infusion significantly increased the aortic diameter and induced a haemorrhage in AAA, while Treg treatment reduced it (*P* < 0.05, Figure 1B and D). These results verified previous studies that Tregs protected against the development of AAA.

### 3.2 Tregs transfer attenuated COX‐2 expression in mice

**Figure 2:**
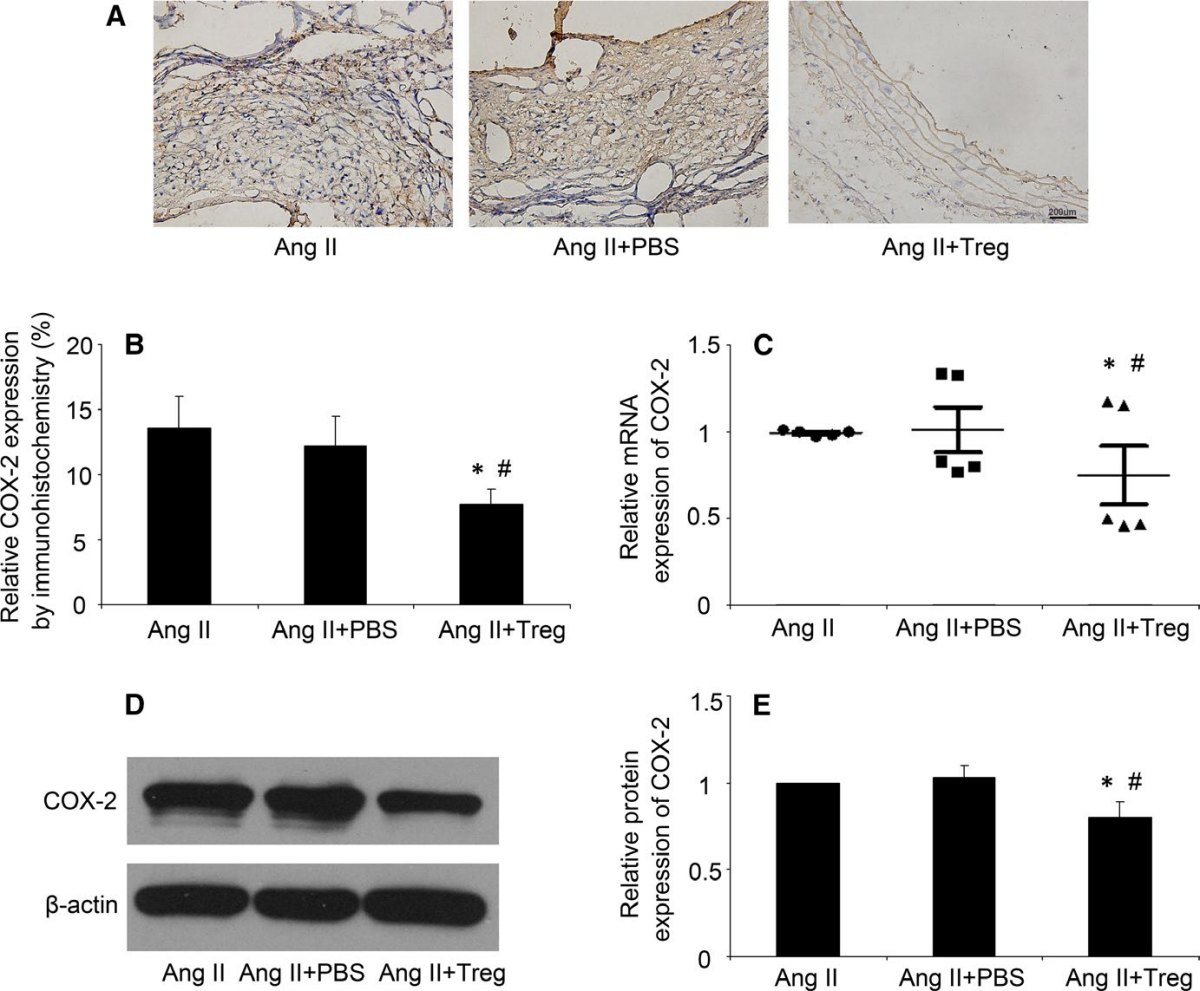
Tregs reduced the COX‐2 expression in Ang II‐infused ApoE^−/−^ mice. A, Representative immunostaining of COX‐2 in three groups of mice infused by Ang II; Bar = 200 µm; B, Quantitative analysis of positive COX‐2 staining. C, Quantitative analysis of mRNA expression of COX‐2; D and E, Western blot analysis of protein expression of COX‐2 in mice. Ang II, angiotension II. ^*^ *P* < 0.05 vs AngII group, ^#^ *P* < 0.05 vs AngII + group

It has been reported that COX‐2 mRNA expression was significantly increased in Ang II‐induced AAA mice compared to the control mice.^19^ In order to clarify the potential effect of Tregs on COX‐2 expression in aortic tissues, immunohistochemical staining was performed in the in vivo experiment. Notably, compared with the control and PBS groups, COX‐2 expression was markedly attenuated in Tregs group (*P* < 0.05, Figure 2A and B). Moreover, the RT‐PCR and Western blot analysis also revealed that Tregs treatment significantly reduced the mRNA and protein expression of COX‐2 in aortic tissues (*P* < 0.05, Figure 2C‐E).

### 3.3 Tregs treatment reduced COX‐2 expression in SMCs and macrophages in mice

**Figure 3:**
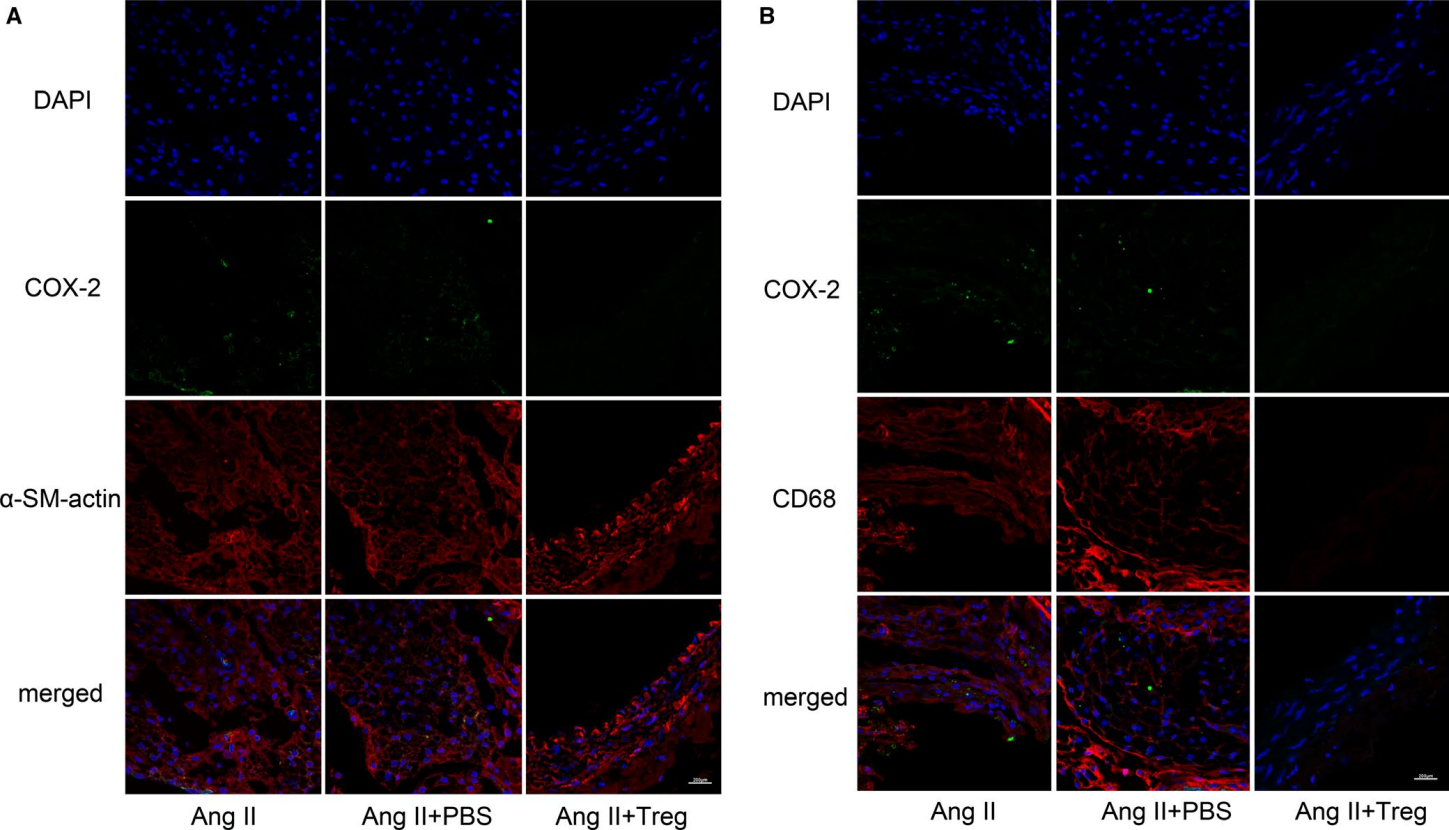
Tregs reduced COX‐2 expression in SMCs and macrophages in mice. A, COX‐2 expression was co‐locolized with the SMCs by immunofluorescence; B, COX‐2 expression was co‐locolized with the macrophages by immunofluorescence; α‐SM actin (red), a biomarker of SMCs; CD68 (red), a biomarker of macrophages. Bar = 200 µm. Ang II, angiotension II

To show the COX‐2 expression in the SMCs or macrophages, immunofluorescence was performed in mice. As shown in Figure 3A, the COX‐2 expression was co‐localized with the SMCs, while there was seldom COX‐2 expression in the Ang II + Treg group (Figure 3A). Meanwhile, Figure 3B showed that there was COX‐2 expression in macrophages in the Ang II group and the Ang II + PBS group, while Treg treatment significantly reduced the expression of COX‐2 (Figure 3B).

### 3.4 Tregs treatment reduced COX‐2 and PGE_2_ expression in cells

**Figure 4:**
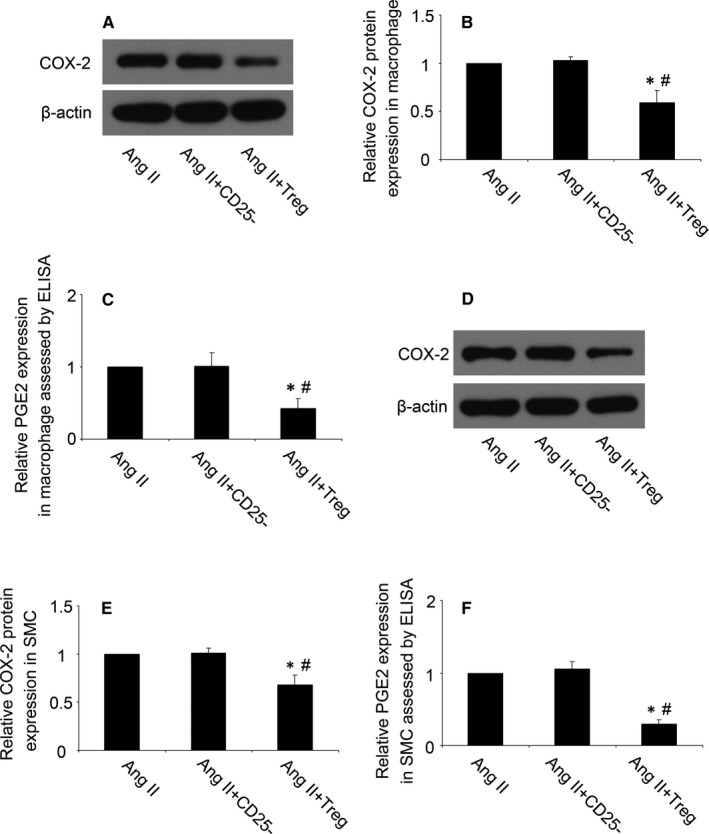
Tregs reduced the COX‐2 and PGE_2_ expression in vitro. A and B, Western blot analysis of protein expression of COX‐2 in Ang II‐stimulated macrophages; C, PGE_2_ expression was assessed by ELISA in macrophages; D and E, Western blot analysis of protein expression of COX‐2 in Ang II‐stimulated mouse smooth muscle cells (SMCs); F, PGE_2_ expression was assessed by ELISA in SMCs. Ang II, angiotensin II; PGE_2_, prostaglandin E_2_. ^*^ *P* < 0.05 vs Ang II group, ^#^ *P* < 0.05 vs Ang II + CD25^−^ group

Cyclooxygenase‐2 can be induced by inflammatory stimuli, such as Ang II.^9^ In endothelial cells, COX‐2 expression was significantly increased after exposure to Ang II.^20^, ^21^ Prostaglandin E_2_ (PGE_2_), synthesized by macrophages and SMCs, increases the production of matrix metalloproteinases (MMPs) and leads to the degradation of the vascular wall.^22^ Prior study has shown that macrophage and SMCs may be the major cells targeted by Tregs in the treatment of AAA.^15^ Therefore, macrophages and SMCs were treated with Ang II after Tregs in our experiment. In the macrophages, we found that the expression of COX‐2 and PGE_2_ was markedly reduced by Tregs treatment as compared with the control group, while CD4^+^CD25^−^ T cells had no effect on COX‐2 and PGE_2_ expression (*P* < 0.05, Figure 4A‐C). Meanwhile, compared to the control group, Tregs treatment also significantly decreased the COX‐2 and PGE_2_ expression in SMCs (*P* < 0.05, Figure 4D‐F). The results showed that Tregs therapy reduced Ang II‐induced COX‐2 and PGE_2_ expression both in macrophages and SMCs.

### 3.5 Tregs increased SMC viability and induced transition of macrophages phenotype

**Figure 5:**
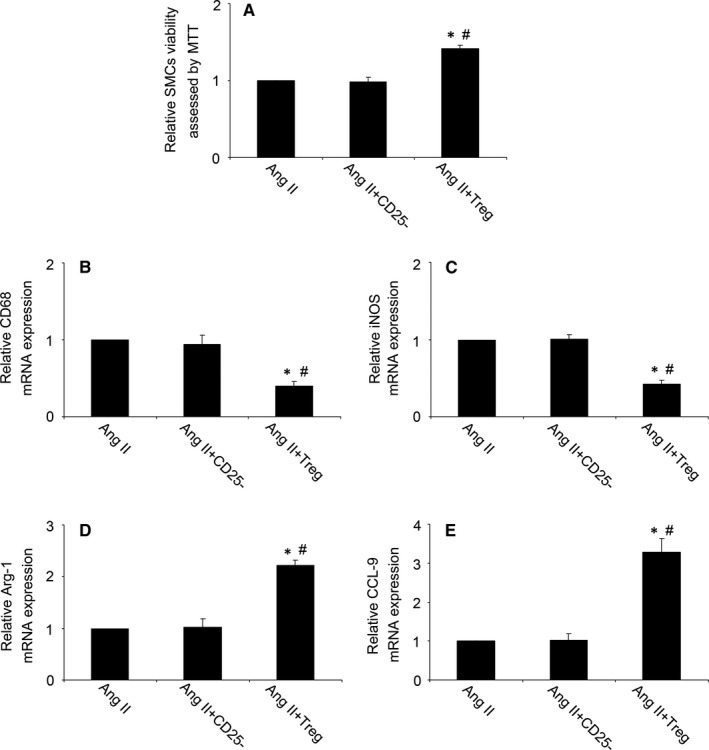
Tregs increased SMC viability and induced transition of macrophages phenotype from M1 to M2. A, Quantitative analysis of SMC viability assessed by MTT; B‐E, Quantitative analysis of mRNA expression of CD68, iNOS, Arg‐1 and CCL‐9 in macrophages. iNOS, inducible nitric oxide synthase; Arg‐1, arginase‐1; CCL‐9, chemokine ligand‐9; Ang II, angiotension II. ^*^ *P* < 0.05 vs Ang II group, ^#^ *P* < 0.05 vs Ang II + CD25^−^ group

Then, we investigated the effect of Treg on the SMC viability and macrophages phenotype. As showed in Figure 5A, compared with the Ang II group, Treg treatment increased the viability of SMCs, while CD4^+^CD25^−^ T cells had no effect. Moreover, RT‐PCR showed that Tregs treatment induced the transition of macrophages phenotype, with the down‐regulation of M1‐related genes (CD86 and iNOS) and up‐regulation of M2‐related genes (Arg‐1 and CCL‐9) (*P* < 0.05, Figure 5B‐E). These results suggested that Tregs increased SMC viability and induced transition of macrophages phenotype from M1 to M2.

## 4 DISCUSSION

Abdominal aortic aneurysm (AAA) is a degenerative disease, which predominantly affects elderly men and leads to a sudden death.^1^, ^23^ AAA is characterized by adventitial and medial cell infiltration of macrophages, destruction of the extracellular matrix and neovascularization.^4^ Unfortunately, the pathogenesis of AAA is still poorly understood. In the experiment, Ang II infusion could successfully induce the AAA formation in ApoE^−/−^ mice and adoptive transfer of Tregs prevented the development of AAA. Meanwhile, we also found that Tregs transfer could suppress the expression of COX‐2 in aneurysmal lesions and macrophages as well as SMCs, and Tregs reduced Ang II‐induced PGE_2_ secretion in vitro. Moreover, Tregs increased SMC viability and induced transition of macrophages phenotype from M1 to M2. Tregs could protect against AAA through inhibition of COX‐2.

Chronic inflammation of the aortic wall, as a prominent pathological feature of AAA, has been considered a fundamental mechanism of this disease.^24^ Aneurysmal segments are characterized by magnified inflammatory response with enhanced infiltration of inflammatory cells and overexpression of pro‐inflammatory cytokines, which contribute to tissue destruction and eventual weakening of vessel wall.^24^ Ang II, as a main hormone of the rennin‐angiotensin system, significantly promoted inflammatory responses, induced matrix degradation and vascular remodelling in ApoE^−/−^ mice.^25^ It has been demonstrated that Tregs reduced the inflammatory response through down‐regulation of inflammatory cell accumulation (mainly macrophage and T cells) and pro‐inflammatory cytokines expression (including MCP‐1, IL‐6 and ICAM‐1) in aortic tissues, which was considered to be a central mechanism of the protection of Tregs.^16^ Moreover, Tregs suppressed Ang II‐mediated vascular injury partly through its anti‐inflammatory characteristics.^25^

Prostanoids are important inflammatory mediators which are associated with aneurysm formation and expansion.^26^ COX‐2 is primarily responsible for prostanoids synthesis, and COX‐2 deletion decreased the expression of PGE_2_ and attenuated inflammatory responses.^11^ Ang II, as a potent inducer of pro‐inflammatory cytokines, induced endothelial COX‐2 expression, which in turn mediated the generation of pro‐inflammatory cytokines.^21^, ^27^ It has been reported that COX‐2 activity and expression maybe play a primary role in the formation and expansion of AAA.^2^ Compared to health donors, COX‐2 expression is obviously increased in AAA patients.^28^ On the contrary, the inhibition of COX‐2 depressed the increased vasoconstrictor response and endothelial dysfunction induced by Ang II infusion.^29^ Moreover, COX‐2 deficiency significantly decreased the incidence and severity of Ang II‐induced AAA in mice models.^2^ In this study, adoptive transfer of Tregs significantly suppressed the mRNA and protein expression of COX‐2 in aortic tissues, suggesting that Tregs protected against AAA probably through the suppression of COX‐2. Immunofluorescence staining showed that the COX‐2 expression was co‐localized with the SMCs and macrophages in AAA, while Tregs treatment could reduce COX‐2 expression in both SMCs and macrophages.

Macrophages produced pro‐inflammatory cytokines, secreted cathepsins and degraded wall elastin, which were considered as key initiating factors in the progress of AAA.^24^ Activated macrophages in the aneurysm were major sources of COX‐2 expression.^2^ Previous study has found that COX‐2 expression was markedly enhanced by Ang II stimulation in macrophages.^30^ In addition, deficiency of COX‐2 attenuated macrophage infiltration and pro‐inflammatory cytokines expression in Ang II‐infused mice.^31^ In our in vitro experiment, macrophages were stimulated by Ang II, and then, COX‐2 expression was assessed by Western blot analysis, and PGE_2_ secretion was analysed by ELISA, which showed that Ang II increased COX‐2 and PGE_2_ expression in macrophages, while Tregs treatment abrogated the increased expression of COX‐2 and PGE_2_ induced by Ang II. Macrophages exist in either a pro‐inflammatory (M1) or anti‐inflammatory (M2) state.^32^ M1 macrophages are characteristically described by their release of pro‐inflammatory cytokines, such as TNF‐ɑ and IL‐1β. Previous studies have demonstrated an increase in M1 macrophages in human AAA tissue.^33^ In contrast, M2 macrophages are considered anti‐inflammatory. The ratio of M1/M2 macrophages plays a critical role in the inflammatory response.^34^ In the experiment, we found that Tregs treatment induced transition of macrophages phenotype from M1 to M2, which suggested that Tregs plays an anti‐inflammatory effect in AAA.

Smooth muscle cells are the active player of extracellular matrix metabolism, and the apoptosis of vascular SMCs contributes to aortic wall damage and lumen dilatation.^35^ Ang II is demonstrated to induce an inflammatory response in SMCs and promotes the apoptosis of vascular SMCs.^15^ In rat vascular SMCs, Ang II stimulation marked increased the mRNA expression of COX‐2.^36^ COX‐2 expression was abundant in medial SMCs of the abdominal aorta in AAA mice and enhanced COX‐2 expression in SMCs contributed to AAA formation by increasing inflammatory cells infiltration.^31^ Therefore, SMCs were also investigated to assess the effect of Tregs on COX‐2 expression in our experiment. We found that Tregs treatment reduced Ang II‐induced COX‐2 protein expression and PGE_2_ in SMCs. Moreover, Tregs treatment increased the viability of SMCs, which might be benefit for the remodelling of the abdominal aorta.

Matrix metalloproteinases (MMPs), as the predominant proteinases in aortic wall, were significantly increased in aneurysmal tissues and mediated the degradation of the extracellular matrix (ECM) and vascular remodelling.^37^ Excessive degradation of ECM causes the destruction of aortic wall, which has been considered as main pathological features of human AAA.^38^ Meanwhile, deficiency of MMPs prevented AAA formation in mice models.^37^ The increased expression of MMPs by Ang II was highly dependent on COX‐2 activation.^39^ In macrophage, PGE2 can also increase the activity and expression of MMP‐2 and MMP‐9.^40^ Therefore, the inhibition of COX‐2 may provide a benefit for the suppression of MMPs activity and contribute to attenuate extracellular matrix destruction and tissue disintegration, which need our further investigation.

In the present study, our results showed that Tregs treatment dramatically decreased the expression of COX‐2 both in vivo and in vitro, suggesting that Tregs protected against AAA formation through inhibition of COX‐2 expression.

## CONFLICT OF INTEREST

The authors declared no conflicts of interest.

## AUTHOR CONTRIBUTIONS

Meng X and Qin W designed the experiments; Liu B, Kong J and An G performed the experiments; Zhang K wrote the paper.

## Data Availability

The datasets used and analysed during the current study are available from the corresponding author on reasonable request.
